# Deaths Attributable to Diabetes in the United States: Comparison of Data Sources and Estimation Approaches

**DOI:** 10.1371/journal.pone.0170219

**Published:** 2017-01-25

**Authors:** Andrew Stokes, Samuel H. Preston

**Affiliations:** 1 Department of Global Health and Center for Global Health and Development, Boston University School of Public Health, Boston, Massachusetts, United States of America; 2 Department of Sociology and Population Studies Center, University of Pennsylvania, Philadelphia, Pennsylvania, United States of America; Hunter College, UNITED STATES

## Abstract

**Objective:**

The goal of this research was to identify the fraction of deaths attributable to diabetes in the United States.

**Research Design and Methods:**

We estimated population attributable fractions (PAF) for cohorts aged 30–84 who were surveyed in the National Health Interview Survey (NHIS) between 1997 and 2009 (N = 282,322) and in the National Health and Nutrition Examination Survey (NHANES) between 1999 and 2010 (N = 21,814). Cohort members were followed prospectively for mortality through 2011. We identified diabetes status using self-reported diagnoses in both NHIS and NHANES and using HbA1c in NHANES. Hazard ratios associated with diabetes were estimated using Cox model adjusted for age, sex, race/ethnicity, educational attainment, and smoking status.

**Results:**

We found a high degree of consistency between data sets and definitions of diabetes in the hazard ratios, estimates of diabetes prevalence, and estimates of the proportion of deaths attributable to diabetes. The proportion of deaths attributable to diabetes was estimated to be 11.5% using self-reports in NHIS, 11.7% using self-reports in NHANES, and 11.8% using HbA1c in NHANES. Among the sub-groups that we examined, the PAF was highest among obese persons at 19.4%. The proportion of deaths in which diabetes was assigned as the underlying cause of death (3.3–3.7%) severely understated the contribution of diabetes to mortality in the United States.

**Conclusion:**

Diabetes may represent a more prominent factor in American mortality than is commonly appreciated, reinforcing the need for robust population-level interventions aimed at diabetes prevention and care.

## Introduction

The prevalence of diabetes has been rising rapidly throughout the world. Global age-standardized diabetes prevalence increased from an estimated 4.3% in 1980 to 9.0% in 2014 in men, and from 5.0% to 7.9% in women.[1] The United States is no exception to this trend. Using combined criteria of self-reported diagnosis, fasting plasma glucose and hemoglobin A1c, the prevalence of diabetes among adults aged 20+ rose from 8.4% in 1988–94 to 12.1% in 2005–10.[2, 3] Trends are similar when HbA1c is the sole criterion.[4, 5] The prevalence of self-reported diagnoses rose very rapidly between 1990 and 2008 and slowly during the 1980’s and between 2008 and 2012.[6]

Diabetes is associated with many diseases and disabilities, including ischemic heart disease, renal disease, visual impairment, peripheral arterial disease, peripheral neuropathy, and cognitive impairment.[7, 8] It is also associated with mortality.[9] In 2010, diabetes was the seventh leading cause of death in the United States. It was listed as the underlying cause of death on 69,091 death certificates (2.8% of total deaths) and appeared in some location on a total of 234,051 death certificates.[10]

The frequency with which diabetes is listed as the underlying cause of death is not a reliable indicator of its actual contribution to the national mortality profile. The sensitivity and specificity of death certificate assignments of diabetes as an underlying cause of death are low, far below those of administrative records or surveys.[11, 12] People who die with diabetes typically have other conditions that may contribute to death. When both diabetes and cardiovascular disease are mentioned on a death certificate, whether or not diabetes is listed as the underlying cause is highly variable and to some extent arbitrary. For example, it is affected by the decedent’s race and sex, whether the death occurs in a hospital, and the number of cardiologists per capita in the area.[13]

An alternative means of estimating the contribution of diabetes to the national mortality profile is to use nationally representative cohorts to identify the excess mortality risk among people with diabetes. That excess risk can be used in combination with the prevalence of diabetes among deaths to estimate the fraction of deaths that would not have occurred in the absence of diabetes. This figure is typically referred to as the population attributable fraction (PAF).[14] Saydah et al.[15] used this approach for individuals aged 30–75 who were surveyed in the National Health and Nutrition Examination Survey II (NHANES II) between 1976 and 1980. They concluded that diagnosed diabetes was responsible for 3.6% of deaths. If undiagnosed diabetes were included, the PAF rose to 5.1%.

These estimates were based on the prevalence of diabetes during 1976–80 and do not account for the subsequent upsurge in prevalence. Furthermore, the relative risks of death associated with diabetes may have declined over time.[16, 17] In this paper, we use NHANES and the National Health Interview Survey (NHIS) to estimate the fraction of deaths attributable to diabetes for cohorts aged 30–84 during the period 1997–2011. We compare estimates of the fraction of deaths attributable to diabetes to comparable measures based on national vital statistics.

## Methods

We estimated the mortality consequences of diabetes in two nationally representative samples of US adults surveyed in the National Health and Nutrition Survey (NHANES) and in the National Health Interview Survey (NHIS). In both data sets, individuals were linked to deaths in the National Death Index through December 31, 2011, the last date to which the National Center for Health Statistics has performed this linkage. Although NHIS only provides self-reports of the presence of diabetes, it has the advantage of a much larger sample size, allowing us to examine how diabetes’ contribution to mortality varies with certain characteristics. NHANES contains data both on self-reported diabetes and on HbA1c levels, a preferred biomarker for the presence of diabetes. Drawing on both data sources provides a more comprehensive picture of the contribution of diabetes to deaths in the United States than using either source alone.

The NHIS is an annual cross-sectional survey of the non-institutionalized U.S. population. We pooled those surveyed from 1997 to 2009. NHIS data were obtained through the Integrated Health Interview Series, which is a publicly available set of harmonized NHIS variables.[18] NHIS assessed diabetes status by asking participants whether a doctor or other health professional had ever told them that they had diabetes. While less precise than clinical measures, self-reports of diabetes have high sensitivity and specificity. In a review of 12 studies of self-reports, the median sensitivity (proportion of cases correctly identified) was 81% and median specificity (proportion of non-cases correctly identified) was 99%.[11] Response categories for the self-reported diabetes question included yes/no as well as “borderline”. We considered individuals in the latter group as non-diabetic for purposes of this study.

We adopted several inclusion criteria in the analysis of NHIS data. We restricted the sample to individuals between the ages 30–84 at the time of survey with non-missing data on diabetes, mortality status and model covariates. Our analysis begins with individuals at age 30 because the incidence of diabetes and of mortality is very low at younger ages. The age range terminates at age 85 because the multiple pathologies typically present above age 85 makes the role of diabetes increasingly ambiguous. Second, we limited mortality follow-up to five years beyond the survey date in order to reduce the length of time between diagnosis and exposure to death. The sample size for analyses of the NHIS was 282,322 individuals.

NHANES is a continuous series of nationally representative surveys of the non-institutionalized population of the United States. We used data from NHANES cohorts surveyed from 1999 to 2010. We defined diabetes on the basis of the HbA1c test. Advantages of HbA1c include that it better reflects average glycemia and exhibits greater stability and lower variation within individuals compared to other diagnostic markers, such as fasting plasma glucose. [19] Furthermore, because HbA1c does not require fasting, it is available for all rather than a subset of NHANES participants. We used the American Diabetes Association (ADA) guidelines [19] to classify individuals with diabetes as having an HbA1c value greater than 6.5%. We further classified as diabetic persons whose HbA1c values were below 6.5% but who reported use of an oral hypoglycemic agent or insulin. For the analysis of self-reported diabetes we classified individuals with “borderline” diabetes as non-diabetic to be consistent with the analysis of the NHIS. We excluded individuals with missing information on diabetes, mortality status and model covariates. To create a comparable age range to that used in the NHIS analysis, we censored individuals upon their achievement of age 90.0. The sample size for analyses of the NHANES was 21,814 individuals.

We used information provided on the death certificate to identify whether diabetes was assigned as the underlying cause of death, defined by the World Health Organization as "the disease or injury which initiated the train of events leading directly to death, or the circumstances of the accident or violence which produced the fatal injury." [20] When diabetes was mentioned anywhere on the death certificate, including as underlying cause, we considered diabetes to be a “contributing cause of death”. The frequency of diabetes as an underlying or contributing cause of death is compared to its population attributable fraction (PAF), defined by the World Health Organization “the proportional reduction in population disease or mortality that would occur if exposure to a risk factor were reduced to an alternative ideal exposure scenario.” [21] In the present case, the alternative ideal exposure is the absence of diabetes.

We estimated Cox models relating diabetes status to all-cause mortality with age as the underlying time scale. The preferred model was adjusted for sex, age, race/ethnicity (non-Hispanic white, non-Hispanic Black, Hispanic, Other), educational attainment (less than high school graduate, high school graduate, and more than high school) and smoking status (never, former, current). In a sensitivity analysis, we adjusted for body mass index (BMI) using the categories <18.5, 18.5–25; 25–30; 30–35; and 35+. BMI values are in units of kg/m^2^ and are based on self-reported height and weight in the NHIS and measured height and weight in the NHANES. In stratified analyses, we used a threshold of 30 kg/m^2^ for defining obesity status.

We calculated the proportion of deaths attributable to diabetes (population attributable fraction (PAF)) using the following formula:

$$PAF=\overset{k}{\underset{i=0}{\sum }}pd_{i}\left(\frac{HR_{i}-1}{HR_{i}}\right)$$ (1)

where pd_i_ refers to the proportion of decedents in diabetes category *i* and HR_i_ refers to the hazard ratio with respect to mortality for an individual in category *i*.[22] Those without diabetes were assigned a hazard ratio of 1.00. The proportion of deaths occurring to those with diabetes, as well as the hazard ratios, were based on estimates specific to the group for whom PAF values were provided.

The proportional hazards assumption was tested using a time-varying coefficients model. Because the interaction term between attained age and diabetes status was significant in this model, indicating a violation of proportionality, we performed a sensitivity analysis in which we re-estimated PAF values using age-specific hazards obtained from the time-varying coefficients model. This was accomplished by applying Eq 1 to data in 5-year wide age intervals using the predicted hazard ratio at the midpoint of each interval. To estimate the PAF value for all ages combined, we weighted the age-specific PAF values by the age distribution of deaths for that group.

In calculating hazard ratios, prevalence values and PAFs, we adjusted for unequal probabilities of selection and non-response using sample weights and accounted for the complex survey design. All analyses were performed using STATA 13 (StataCorp, Texas, USA). We estimated variances with the SVY routine, which uses Taylor series linearization. Uncertainty intervals for population attributable fractions were estimated using the punafcc package.[23]

## Results

Table 1 shows the characteristics of individuals surveyed in NHANES and NHIS. The total sample size was more than 10 times larger in NHIS than in NHANES. The distribution of characteristics was very similar in the two data sources with the exception of BMI, which was based on self-reported weight and height in NHIS, whereas it was measured in NHANES. Consistent with a well-documented tendency for people to underestimate their weight and overestimate their height [24], the proportion obese was higher in NHANES (35.0%) than in NHIS (28.5%).

**Table 1:** Characteristics of the NHIS and NHANES samples, adults ages 30–84.

	NHIS (n = 282,322)		NHANES (n = 21,814)	
	n	%	n	%
Sex				
Male	123,584	47.9	10,849	48.3
Female	158,738	52.1	10,965	51.7
Age at baseline				
30–59	199,022	73.3	13,349	73.6
60–74	57,881	19.2	6,306	20.1
75–84	25,419	7.5	2,159	6.2
Race/ethnicity				
Non-Hispanic White	188,990	74.2	10,919	73.3
Non-Hispanic Black	39,411	10.8	4,319	10.5
Hispanic	42,915	10.6	5,787	11.5
Other	11,006	4.4	789	4.8
BMI Category (kg/m2)				
Underweight	4,209	1.4	273	1.4
Normal Weight	96,829	33.8	5,570	28.4
Overweight	100,975	36.2	7,876	35.2
Obese I	45,588	16.3	4,689	20.2
Obese II	34,721	12.2	3,406	14.8

BMI: body mass index; Height and weight data for calculating BMI were self-reported in the NHIS and measured in the NHANES. Percentage values were calculated using sample weights. Sources: The NHIS sample includes data for years 1997–2009; the NHANES sample includes data for years 1999–2010.

**Fig 1:**
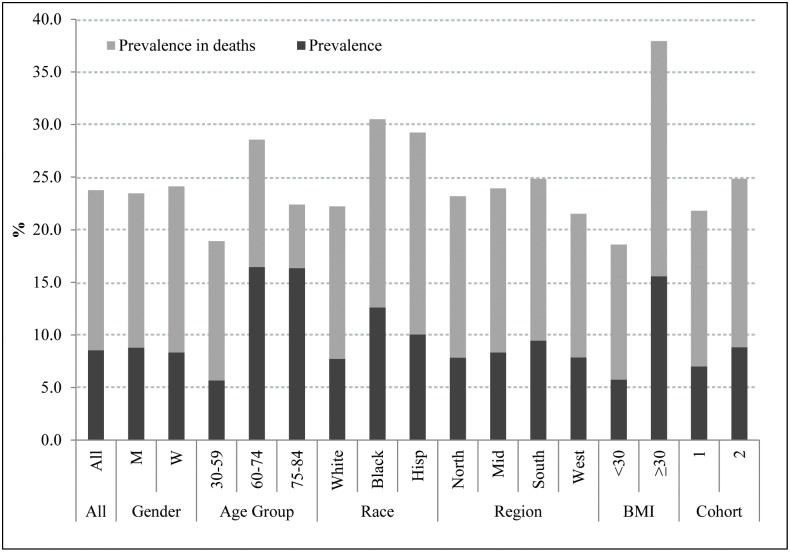
Prevalence of diabetes in the sample and among individuals who died in the total NHIS sample and in various population subgroups. BMI: body mass index. Cohort 1 includes years 1997–2001 and cohort 2 includes years 2002–2006. Source: NHIS.

Fig 1 shows the prevalence of diabetes in NHIS among the surveyed population and among subsequent deaths to those surveyed. In all cases, the prevalence was higher among deaths than among individuals at survey, reflecting the higher mortality of individuals with diabetes. For all groups combined, diabetes had been diagnosed among 8.5% of the population at baseline and among 23.7% of those who died during the 5-year follow-up period. We are not aware of previous estimates that document the very high proportion of individuals dying in the United States who have been diagnosed with diabetes. That value reached 30.5% among blacks and 37.9% among the obese. The value among the obese was approximately double that among the non-obese (18.6%).

**Fig 2:**
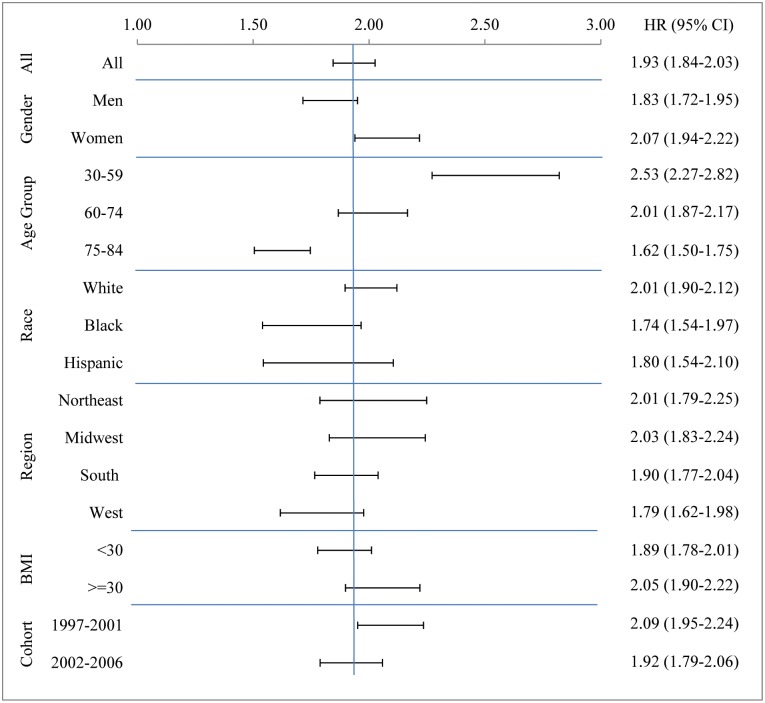
Hazard ratios expressing the association between diabetes status and mortality for all participants and by population subgroup. Source: NHIS years 1997–2009 with prospective mortality follow-up through Dec. 2011.

Fig 2 presents the hazard ratios and their confidence intervals for those diagnosed with diabetes relative to those who were not. For the NHIS sample as a whole, the hazard ratio was 1.93 with a confidence interval extending from 1.84 to 2.03. The hazard ratios were higher in women (2.07, 95% CI 1.94–2.22) compared to men (1.83, 95% CI 1.72–1.95) and for people aged 30–59 (2.53, 95% CI 2.27–2.82) and 60–74 (2.01, 95% CI 1.87–2.17) compared to those aged 75–84 (1.62, 95% CI 1.50–1.75). A decline with age in the hazard associated with diabetes has also appeared in other studies.[9,25]

The hazard ratios in Fig 2 combine with prevalence values shown in Fig 1 to produce estimates of the proportion of deaths attributable to diabetes. These values are presented in Table 2. For the cohort as a whole, an estimated 11.5% of deaths were attributable to diabetes. Among the characteristics examined, by far the highest proportion of deaths attributable to diabetes, 19.4%, occurred among obese people, compared to only 8.8% among the non-obese.

**Table 2:** Percent of deaths attributable to diabetes according to demographic characteristics by method of estimation, NHIS.

	Diabetes assigned as underlying cause			Diabetes assigned as contributing cause			Population attributable fraction		
	%	95% CI		%	95% CI		%	95% CI	
All	3.3	2.9	3.6	10.8	10.2	11.4	11.5	10.5	12.4
Sex									
Male	3.0	2.6	3.5	10.4	9.6	11.2	10.6	9.3	11.9
Female	3.6	3.1	4.0	11.3	10.4	12.2	12.5	11.2	13.8
Age Category									
30–59	3.2	2.5	3.9	9.0	7.9	10.0	11.4	9.7	13.2
60–74	4.0	3.5	4.6	12.8	11.7	13.9	14.4	12.6	16.1
75–84	2.6	2.1	3.0	10.1	9.2	11.1	8.6	7.5	9.6
Race/ethnicity									
NH White	2.6	2.3	3.0	9.7	9.0	10.3	11.1	10.5	11.8
NH Black	5.0	3.9	6.0	14.4	12.6	16.2	13.0	10.8	15.1
Hispanic	7.0	5.3	8.7	15.7	13.3	18.1	13.0	10.5	15.5
Region									
Northeast	3.8	3.0	4.5	10.1	8.9	11.4	11.6	10.3	12.9
Midwest	2.9	2.2	3.6	10.2	8.8	11.5	12.1	10.9	13.3
South	3.3	2.8	3.8	11.0	10.0	12.0	11.7	10.8	12.7
West	3.2	2.4	4.1	11.9	10.3	13.5	9.5	8.3	10.7
BMI (kg/m2)									
<30	2.4	2.1	2.8	8.6	8.0	9.3	8.8	8.2	9.4
≥30	5.6	4.8	6.4	16.7	15.3	18.1	19.4	18.0	20.9
Cohort									
1997–2001	3.6	3.1	4.0	10.9	10.0	11.7	11.4	10.7	12.1
2002–2006	3.2	2.7	3.7	11.0	10.1	12.0	11.9	11.0	12.8

NH: non-Hispanic; BMI: body mass index. Source: NHIS, 1997–2011.

The PAF value was 10.6% for males and 12.5% for females. The female excess was primarily a result of the higher hazard ratio associated with diabetes for females (Fig 2). The PAF for both Blacks and Hispanics was 13.0%, compared to 11.1% for Non-Hispanic Whites. The high PAF for Hispanics and non-Hispanic blacks is entirely a product of a high prevalence of diabetes rather than of higher hazard ratios. As shown in Fig 2, the hazard ratio for Whites was 2.01, compared to 1.74 for Blacks and 1.80 for Hispanics. Likewise, the modest rise in PAF from the cohort surveyed in 1997–2001 to that in 2002–06 is completely a product of rising prevalence.

The proportion of deaths in which diabetes is assigned as the underlying cause of death was much lower than the population attributable fraction (Table 2). For the nation as a whole, only 3.3% of deaths in these cohorts were assigned to diabetes as the underlying cause. However, when deaths were added in which diabetes was mentioned on the death certificate elsewhere than as the underlying cause of death, the proportion rose to 10.8%, similar to the PAF value. The patterns of variation in the two series across subgroups are also correlated, with the highest frequency of death certificate mentions of diabetes also occurring among the obese. It is not the case that attending physicians and coroners are simply reporting on death certificates the presence of diabetes among those who have been diagnosed with the disease; more than twice as many decedents had been diagnosed with diabetes (23.7%) as had the condition reported anywhere on their death certificate (10.8%).

Estimates derived from NHIS and NHANES are compared in Table 3. The hazard ratio using the HbA1c criterion in NHANES was 1.88, with a wide confidence band from 1.63 to 2.16. The estimated value is clearly similar to the estimate of 1.93 derived from NHIS. Could some of the small difference between these estimates be attributed to differences in the measures used to identify diabetes? That question can be addressed by virtue of the two criteria used in NHANES to assess diabetes status. Using only self-reports in NHANES, the hazard ratio was 2.00 (95% CI 1.75–2.28), slightly higher than the estimated hazard ratio of 1.88 using HbA1c and the hazard ratio of 1.93 using self-reports in NHIS.

**Table 3:** Comparison of the percent of deaths attributable to diabetes in the NHIS and NHANES cohorts.

	Prevalence in deaths (%)	HR	PAF (%)	Underlying (%)	Contributing (%)
NHIS	23.7	1.93	11.5	3.3	10.8
	(22.9–24.6)	(1.84–2.03)	(10.5–12.4)	(2.9–3.6)	(10.2–11.4)
NHANES—SR	23.5	2.00	11.7	3.7	12.1
	(21.2–25.7)	(1.75–2.28)	(9.2–14.2)	(2.7–4.7)	(10.1–14.1)
NHANES—M	25.3	1.88	11.8	3.7	12.1
	22.9–27.8	(1.63–2.16)	(9.0–14.6)	(2.7–4.7)	(10.1–14.1)

HR: hazard ratio; PAF: population attributable fraction. Source: The NHIS sample includes data for years 1997–2009; the NHANES sample includes data for years 1999–2010. Individuals in both cohorts were followed prospectively for mortality through Dec. 2011.

The multiple criteria for diabetes in NHANES enable us to distinguish between those with diabetes who report a diagnosis of diabetes and those whose diabetes is undiagnosed. One might imagine that those with undiagnosed diabetes were at higher risk of death precisely because it is undiagnosed. However, individuals with diagnosed diabetes had significantly elevated risks (HR 2.05, 95% CI 1.77–2.37), whereas the risk associated with undiagnosed diabetes was weaker and not significant (HR 1.28, 95% CI 0.95–1.74). It appears that those whose diabetes is undiagnosed have, on average, a better prognosis than those whose diabetes is diagnosed (see also [26,27]).

Table 3 also shows that the proportion of deaths attributable to diabetes among cohorts aged 30–84 when surveyed in NHANES was 11.8% using the HbA1c criterion and 11.7% based on self-reports. These values are very close to the value of 11.5% estimated using NHIS and the confidence intervals overlap substantially. The proportion of deaths in which diabetes is listed as the underlying cause, or mentioned anywhere on the death certificate, was also very similar in NHANES and NHIS (Table 3).

## Discussion

The study most comparable to ours used cohorts aged 30–74 who were surveyed in NHANES II between 1976 and 1980 and followed them into mortality statistics through 1992. (15) Using self-reported diabetes, this study reported a hazard ratio of 1.9, a prevalence at survey of 4.3%, and a PAF of 3.6%. Adding undiagnosed cases that were detected using fasting plasma glucose, the PAF increased to 5.1%. The main reason why the PAF values in the present study are much higher is that the prevalence of diabetes has risen sharply since 1976–80 (see also [28,29]).

Our estimates of the hazard ratios associated with diabetes are similar to those found in other studies. Gregg et al.[30] also used NHIS data to estimate hazard ratios (HRs) for adults aged 18+ with self-reported diabetes. They found hazard ratios in the range of 1.68–2.13 for males and females in data for cohort surveyed in 1997–98, 1999–2000, and 2001–03, followed by a fall off to 1.52–1.58 in 2003–04. We also find a decline between cohorts surveyed in 1997–2001 and 2002–06 but it is less abrupt, perhaps because our cohorts pertain to time periods that are twice as wide. The Gregg et al. mortality analysis extended only to 2006 while our mortality analysis extends to 2011. The DECODE study pooled 20 European studies including 30,000 people with ages ranging from 30 to 89.[31] Using fasting plasma glucose criteria, the relative risk of death for those with diabetes was approximately 1.9–2.0.[27, 31] Using a variety of clinical diagnostic criteria, a British cohort study of 44,000 individuals aged 35–89 reported a hazard ratio of 1.93.[25] Our estimated hazard ratios are consistent with these studies.

We explored the sensitivity of our results to three alternative specifications. First, we implemented a time-varying coefficients model using data from the NHIS to investigate whether introducing an interaction term between attained age and diabetes status in the hazard model would influence estimates of the overall PAF. The resulting PAF value for females was 12.9% compared to 12.5% without age interactions. For males, the corresponding values were 11.0% and 10.6%. Thus, the introduction of age interactions has small effects on the estimated PAF values, effects that are well within the confidence intervals shown in Table 2.

Second, we introduced a five-category control for body mass index (BMI) in our regression used to estimate the hazard ratio for diabetes. We anticipated that introducing obesity may lower the PAF because diabetes status, positively correlated with obesity, may be associated with the higher mortality suffered by obese people from cardiovascular disease. Such an association would spuriously inflate the hazard ratio for diabetes. Instead, the hazard ratio estimated on NHIS data rose from 1.93 to 2.00 when obesity was controlled and the PAF value rose from 11.5% to 11.9%

Third, we investigated how the PAF value would change if pre-diabetes were included in the analysis. For this purpose, we used values of HbA1c in NHANES to define the categories of normal (less than 5.7%), pre-diabetic (5.7–6.4%) and diabetic (6.5% and above). People with an HbA1c value below 6.5% with reported use of oral glycemic medication or insulin continued to be included in the diabetic category. The PAF value using this three-category variable is 14.0% (95% CI 9.3–18.5), compared to the earlier estimate of 11.8% (95% CI 9.0–14.6) when considering diabetes as a dichotomy. In this paper, we treat diabetes as a dichotomy, a status that is either achieved or is not. This approach enables us to use the rich resources of both NHIS and NHANES in the investigation. But it should be borne in mind that, if we were instead to treat diabetes as a disease process identified by above-normal HbA1c levels, then the proportion of deaths attributable to it would be higher by approximately 2.2%.

The major strength of this analysis is its use of two national data sources and two different measures of diabetes to investigate its role in American mortality. That these data sources and measures produce similar estimates of the proportion of adult deaths attributable to diabetes gives greater confidence in each of the separate estimates.

A weakness of the present approach is that diabetes status is measured at baseline and could change during the follow up period. While transitions from diabetes to non-diabetes status are rare, transitions from non-diabetes to diabetes are not rare. To mitigate this potential source of measurement error we restricted mortality follow-up to 5 years. The annual incidence rate of diabetes among non-diabetic adults is approximately 0.008.[6] That means that, over the 5-year follow-up period, an average of 2% [.008 x 2.5] of those without the disease at the outset would have developed it. These new cases represent a misclassification error that is likely to induce a slight downward bias in our estimates of the mortality effects of diabetes and the fraction of deaths attributable to it.[32]

## Conclusion

To investigate the proportion of deaths attributable to diabetes, we used two independent data sets and two different criteria for identifying diabetes among individuals aged 30–84. We found a high degree of consistency in the resulting hazard ratios, estimates of diabetes prevalence, and estimates of the proportion of deaths attributable to diabetes. The proportion of deaths attributable to diabetes was estimated to be 11.5% using self-reports in NHIS, 11.7% using self-reports in NHANES, and 11.8% using HbA1c in NHANES. The proportion of deaths attributable to diabetes is much greater than the 3.3–3.7% of deaths in which diabetes is assigned as the underlying cause of death.

Responsibility for approximately 12% of deaths would make diabetes the third leading cause of death in the United States in 2010, after diseases of the heart and malignant neoplasms and ahead of chronic lower respiratory diseases and cerebrovascular diseases.[33] The inclusion of pre-diabetes in the risk category would raise the proportion of deaths attributable to diabetes by an additional 2%. These results demonstrate that diabetes is a major feature on the landscape of American mortality and reinforce the need for robust population-level interventions aimed at diabetes prevention and care.
